# Evaluation of the effectiveness and efficiency of the East African community joint assessment procedure by pharmaceutical companies: Opportunities for improvement

**DOI:** 10.3389/fphar.2022.1031289

**Published:** 2022-11-03

**Authors:** Nancy Ngum, Jane Mashingia, Margareth Ndomondo-Sigonda, Stuart Walker, Sam Salek

**Affiliations:** ^1^ Department of Clinical and Pharmaceutical Sciences, School of Life and Medical Sciences, University of Hertfordshire, Hatfield, United Kingdom; ^2^ African Union Development Agency—New Partnership for Africa’s Development (AUDA-NEPAD), Johannesburg, South Africa; ^3^ East African Community Secretariat, Arusha, Tanzania; ^4^ Centre for Innovation in Regulatory Science, London, United Kingdom; ^5^ Institute of Medicines Development, Cardiff, United Kingdom

**Keywords:** EAC joint assessment procedure EAC-MRH, effectiveness, efficiency, applicants, common technical documents, joint assessment procedure, pharmaceutical companies

## Abstract

**Background:** A 2021 study to determine the viewpoints among the seven member countries regarding the effectiveness (i.e., achieving the intended outcomes) and efficiency (i.e., achieving the intended outcomes in timely manner with the resources available) of the East African Community Medicine Regulatory Harmonisation (EAC-MRH) Joint Assessment Procedure recommended the conduct of a similar study among pharmaceutical company applicants. The aim of this study then was to evaluate the effectiveness and efficiency of the current EAC-MRH operating model from the applicants’ perspective, including the challenges and opportunities for improvement.

**Methods:** Using the Process Effectiveness and Efficiency Rating for Industry questionnaire developed by the authors, data were collected from company representatives responsible for EAC joint procedure submissions.

**Results:** Responses from 14 study participants underlined the support of pharmaceutical companies for the EAC-MRH initiative, which has facilitated the harmonisation of registration requirements across the EAC region leading to one registration for all countries and a reduction of the workload for both applicants and assessors. In addition, it is expected that shorter timelines for approval will lead to improved access to quality-assured essential medicines in the region. Access to various markets at the same time was also noted as an important benefit to pharmaceutical companies. Noted challenges include a lack of process information, a lack of centralised submission and tracking process and a lack of mandated central registration. A key strategy proposed by participants is the establishment of a regional administrative body to centrally receive and track EAC applications and the eventual establishment of a Regional EAC Medicines Authority.

**Conclusion:** This is the first study evaluating the performance of the EAC work-sharing initiative from the point of view of the applicants. In general, the applicants believe that the system performs efficiently and fulfils its promise. However, some participants indicated that in some countries an EAC positive recommendation does not directly result in an individual country approvals. Following the recommendations listed in this report may mitigate identified areas for improvement and facilitate the overall goal of the EAC-MRH initiative to expedite the availability of needed quality-assured medicines to patients in the region.

## 1 Introduction

Countries need fully functional regulatory systems in order to respond to public health needs as well as to enhance access to safe and effective medicines (Kusinitz M et al., 2017). One of the determinants of access to essential medicines is regulatory filing and registration (Sillo et al., 2020). In Africa, regulatory authorities face several challenges in regulating medicines, as most national medicines regulatory authorities (NMRAs) are not adequately resourced when compared with established regulatory authorities. As of 2022, only five NMRAs in Africa, Ghana, Tanzania, South Africa, Egypt and Nigeria have attained the World Health Organization (WHO) maturity level 3 status; that is, a stable, well-functioning regulatory authority (Broojerdi, 2020). Since 2009, the African Union Development Agency (AUDA-NEPAD) has been spearheading the African Medicines Regulatory Harmonisation (AMRH) initiative as a means of improving access to safe, high-quality and effective medicines in Africa through the harmonisation of regulatory requirements (Dansie et al., 2019). Including the East African Community Medicines Regulatory Harmonisation (EAC-MRH) programme, five regional harmonisation initiatives have been established in Africa to increase the number of quality-assured products available to patients, by simplifying the registration processes for manufacturers and improving capacity (Sillo et al., 2020; Ndomondo-Sigonda et al., 2021).

### 1.1 The EAC-MRH initiative

The EAC-MRH initiative is a joint assessment procedure composed of seven NMRAs in the EAC region. These NNMRAs include Burundi Food and Medicines Regulatory Authority (ABREMA), Bujumbura, Burundi; Kenya Pharmacy and Poisons Board (KPPB), Nairobi, Kenya; National Drug Authority (NDA), Kampala, Uganda; Zanzibar Medicines and Medical Devices Agency (ZMDA), Zanzibar, Tanzania; Drug and Food Control Authority (DFCA), Juba, South Sudan; Rwanda Food and Drugs Authority (RFDA), Kigali, Rwanda; and Tanzania Medicines and Medical Devices Authority (TMDA), Dar Es Salaam, Tanzania.

To provide guidance to the NMRAs in managing applications for registration of human medicinal products in the EAC, a compendium was developed in 2014 by the Technical Working Group (TWG) on Medicines Evaluation and Registration (MER) of the EAC-MRH Project. The compendium has five modules and sets out procedures and requirements for the implementation of Pharmaceutical Products Registration through established Common Technical Documents (CTD) within EAC NMRAs. These documents are based on the International Conference on Harmonisation of Technical Requirements for Registration of Pharmaceutical Products for Human use (ICH) guidelines. The aim of the CTD guidelines is “to provide harmonised medicines registration procedures using the CTD in order to improve access to essential medicines for prevention and treatment of priority disease conditions in the East African region” (EAC Secretariat, 2014). According to Sithole et al. (2022), the CTD format has helped to improve work sharing and the harmonisation of registration requirements and joint reviews in Africa.

With the launch of the EAC-MRH programme in March 2012, member countries have made substantial progress in the reduction of timelines for registration of new medicines using the joint review process. The aim of the regional harmonisation project is to minimise barriers to medicine registration and eventually increase the number of products registered within a shorter timeline. Mashingia and others (2020) reported that from 2012 to 2017 registration timelines were reduced from 24 months to 8–12 months for products reviewed using the new joint assessment process. Started in 2015, the EAC initiative has a decentralised structure, with focus on work sharing and reliance. It is composed of a joint assessment of dossiers for medical products submitted by applicants for review and a joint inspection of manufacturing sites by the assessors (Sillo et al., 2020). As outlined by Ngum and associated (2022), this process has nine steps, starting with the submission of an application and ending with approval at a national level, which is expected to occur within 90 days after a positive recommendation is made. As of December 2021, a total of 159 applications have been received, 144 assessed and 79 products recommended for registration through the EAC-MRH joint procedure, with a median time for recommendation to market authorisation between 30 and 90 days (AUDA-NEPAD, 2021).

A study was conducted in 2021 to determine the views of regulators from the seven NMRAs of the EAC-MRH initiative on the effectiveness and efficiency of the work-sharing initiative. One of the recommendations from this study was to conduct a similar study with the applicants, so that there could be a comparison of the benefits and challenges from the point of view of both key stakeholders (Ngum et al., 2022). The aim of this study was, therefore, to evaluate the effectiveness and efficiency of the current operating model of the EAC-MRH initiative from the applicants’ perspective, including the challenges it faces as well as to identify opportunities for improvement.

## 2 Study objectives

The study objectives were to.

• Obtain the views of the applicants of the EAC-MRH initiative about the performance of the programme to date
• Identify the challenges experienced by applicants throughout the life cycle of the EAC-MRH initiative
• Determine the strengths and weaknesses of the initiative
• Identify the ways of improving the performance of the work-sharing programme
• Envisage the strategy for moving forward.

## 3 Methodology

### 3.1 Study participants

From the 34 applicants identified as using the EAC-MRH initiative to submit applications for registration and marketing authorisation, 25 were determined to be eligible for the study; among this group there were 11 non-responses, leading to a 56% response rate. Study participants were distributed into three categories; Generics (foreign); that is, applicants who manufacture generic medicines outside of the EAC region, Generics (local); that is, applicants who manufacture generic medicines within the EAC region, and Innovators; that is, applicants who submitted applications for registration of innovator medicines. During the period of study (2015–2021), there were no local innovators that submitted applications for innovator medicines for registration.

### 3.2 Data collection

Collection of data started in November 2021 and ended in April 2022. The questionnaire was completed by a representative responsible for EAC joint procedure submissions in each company.

### 3.3 Development of the PEER-IND questionnaire

The authors developed a Process Effectiveness and Efficiency Rating for Industry (PEER-IND) questionnaire to identify the views of applicants on the benefits, challenges and suggestions for improving the performance of the EAC-MRH work-sharing initiative. (Supplementary) PEER-IND comprised five parts; Demographics; Benefits of the EAC-MRH initiative; Challenges of the EAC-MRH initiative; Improving the performance (effectiveness and efficiency) of the work-sharing programme and envisaging the strategy for moving forward.

### 3.4 Ethics committee approval

The study was approved by the Health, Science, Engineering and Technology ECDA, University of Hertfordshire, United Kingdom [Reference Protocol number: LMS/PGR/UH/04988].

## 4 Results

For the purpose of clarity, the results are presented in five parts: Demographics; Benefits of the EAC-MRH initiative; Challenges of the EAC-MRH initiative; Improving the performance (effectiveness and efficiency) of the work-sharing programme; and Envisaging the strategy for moving forward.

### 4.1 Part I- demographics

Most respondents, who presented the views of their companies, held roles as head of regulatory affairs in their respective companies, with regulatory experience ranging between 5 and 21 years. The companies that participated in the study were classified according to their product portfolio and location of their manufacturing sites. Eight (58%) were foreign generic pharmaceutical companies, three (21%) were local manufacturers of generics and three (21%) were innovator pharmaceutical companies (Table 1). Of the 144 dossiers/applications assessed as of 31 December 2021, 55% were generics submitted by foreign companies, 22% were new active substances submitted by innovator companies and 23% were generics submitted by the local company.

**TABLE 1:** Pharmaceutical companies participating in study.

Name of company	Generics (foreign)	Generics (local)	Innovator
Intas pharmaceutical limited	✓		
Bayer			✓
Cipla Quality Chemical Industries Limited	✓		
Dafra Pharma GmbH	✓		
Impact RH360	✓		
Laboratoire Aguettant	✓		
Laboratory and Allied Ltd.		✓	
Prodigy Healthcare Limited		✓	
Universal Corporation Limited		✓	
La Renon Healthcare Pvt. Ltd. 9 (India)	✓		
Novartis South Africa			✓
F. Hoffmann-La Roche Ltd			✓
Cipla Ltd.	✓		
Amring Farma SRL, Romania	✓		

### 4.2 The EAC countries in which companies market their products

**FIGURE 1:**
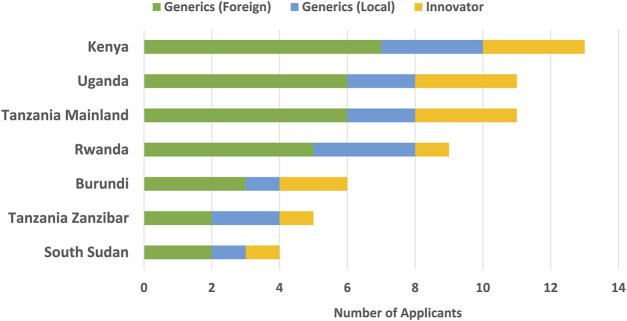
EAC countries in which companies market products.

All the companies indicated they had a separate record of applications submitted for assessment under EAC-MRH to facilitate tracking and adherence to deadlines. The majority of the companies market products in Kenya, Tanzania Mainland and Uganda (Figure 1). The applicants gave various reasons why their companies market products in the selected countries, including the fact that these countries provide excellent and ready market potential for pharmaceutical companies, as wider market coverage maximises revenues and economies of scale. In addition, there is an available patient pool for products in these markets, with market stability and predictability, with an established distribution chain, as well as mature healthcare systems.

Most companies are interested in registering medicines in countries with developed medical systems like oncology and rheumatology centres. The majority of pharmaceutical companies want to ensure maximum reach and access of essential healthcare products to positively impact society and sometimes the marketing of products in these countries is based on partner and donor interest. Companies that are leading manufacturers of essential medicines for high disease burden like antiretrovirals and anti-malarials in the region are marketing medicines and healthcare solutions not only in the EAC member countries, but in the whole of Sub-Saharan Africa.

The capacity of NMRAs in the region is key, as some of the countries have not initiated the process of medicine registration as they do not have fully functional regulatory authorities. Some countries access some medical products through import permits so that marketing in such countries is not required. Aspects such as lack of security, political, and market stability, weak regulatory and healthcare systems, weaknesses in the supply and distribution processes are some reasons why some manufacturing companies do not market products in all EAC countries.

### 4.3 Part II- benefits of the EAC-MRH initiative to regulators and pharmaceutical companies

**FIGURE 2:**
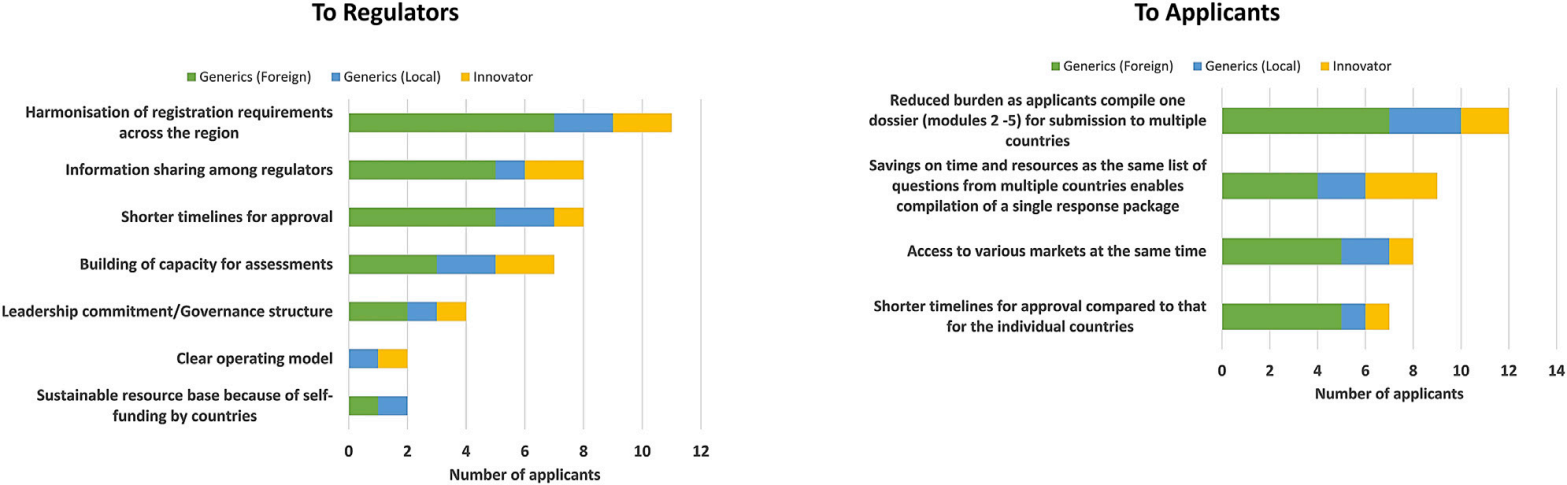
Benefits of the EAC-MRH initiative.

Pharmaceutical companies identified the harmonisation of registration requirements across the region, shorter timelines for approval and information sharing among regulators as well as building capacity for assessments as the top four benefits of the EAC initiative (Figure 2). One registration for all countries was also mentioned as a benefit, leading to access to various markets at the same time. However, it was noted that the shorter approval timelines and clear operating model are currently applicable only for Tanzania.

Several benefits of the initiative were indicated, including reduced burden, as applicants compile one dossier (modules 2–5) for submission to multiple countries, savings in time and resources as applicants receive the same list of questions from multiple countries, which enables the compilation of a single response package. Shorter timelines for approval compared with those for individual countries as well the ability to launch products simultaneously in all markets were also identified (Figure 2).

However, some companies mentioned that they submitted documentation for EAC in August 2019 but did not receive any response from the EAC-MRH Secretariat. Meanwhile, they obtained a national registration for their products based on normal assessment procedure in three countries (Tanzania, Uganda and Kenya). As previously mentioned, others indicated that some of the above benefits are currently applicable only for Tanzania, as the procedure’s benefits declined over time for other countries since an EAC positive opinion does not directly result in approval in those countries. Also, NMRAs often request additional information after an EAC positive opinion, which further delays approval and patients’ access in individual markets.

The applicants are required to apply for a marketing authorization in EAC countries after a joint positive recommendation. However, the time to registration of the product at a country level will depend on when the country specific application is submitted and if additional information is requested by the country. Therefore, the times given for approval represent the time to national approval and not to the time of EAC recommendation. In general, full applications are submitted with only a few abridged dossiers. Most of these applications are for generic products where only quality assessments are conducted. Furthermore, the assessment reports are only from the EAC region. Unfortunately, according to some applicants, their interaction with the EAC procedure has not led to any improvement in product dossier assessment, although their hope is that in the future dossier submission will improve.

Quicker access to quality-assured medicines and increased availability of medicines were the benefits for patients indicated by all applicants, although reduced prices of medicines is not yet an outcome of the initiative for patients.

### 4.4 Part III- challenges of the EAC-MRH initiative

**FIGURE 3:**
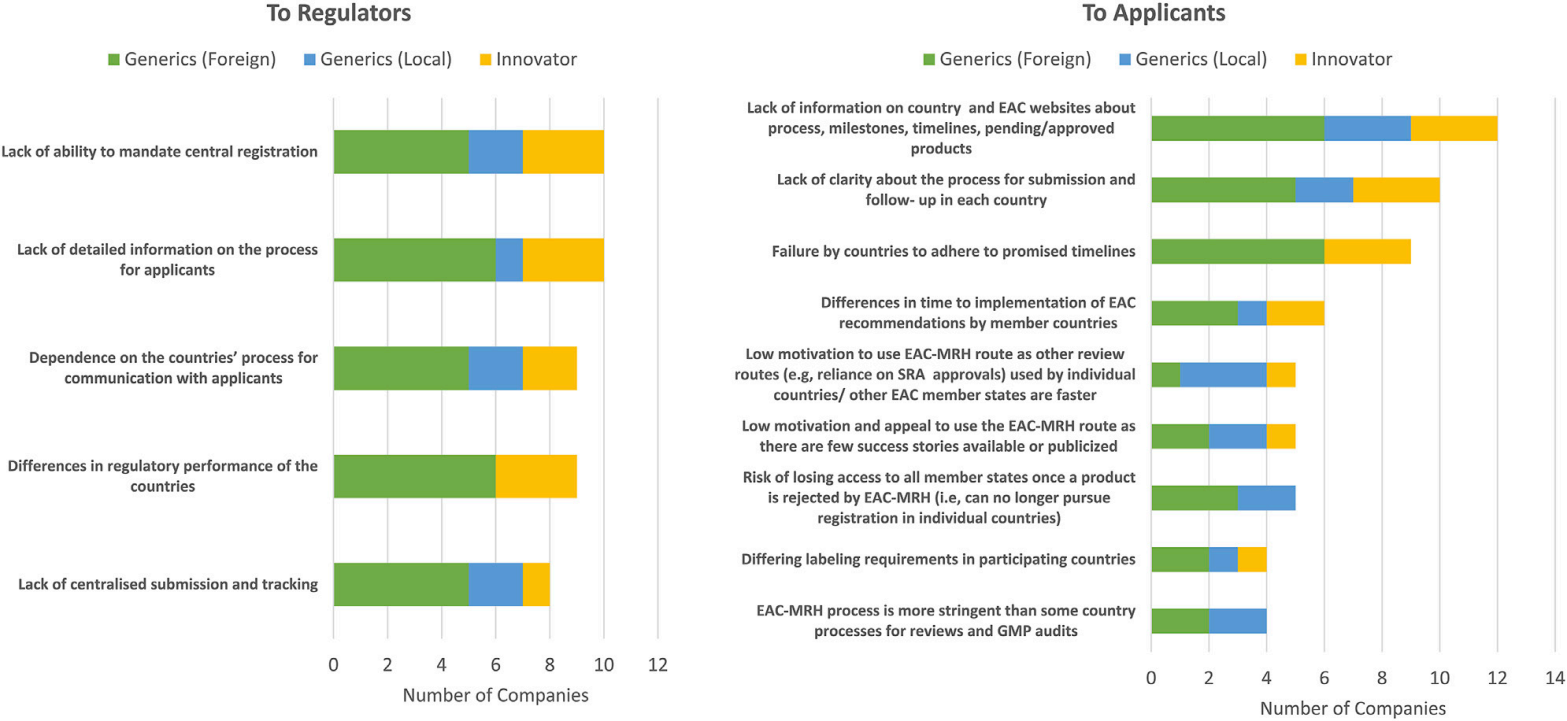
Challenges of the EAC-MRH initiative.

Some of the challenges of the EAC-MRH initiative highlighted were a lack of detailed information on the process for applicants, differences in regulatory performance of the countries, a dependence on the countries’ process for communication with applicants; a lack of centralised submission and tracking processes; an inability to mandate central registration; and an unclear process for obtaining actual marketing authorisation after assessment (Figure 3). Other challenges include the lack of harmonisation between the different EAC member states or harmonisation for variation processes. There is a lack of uniformed and binding requirements for all countries as, although regional guidelines exist, they are not always fully implemented in the national procedures. Also, the presence of country-specific requirements that follow an EAC-MRH positive opinion further delays the approval process.

#### 4.4.1 Challenges faced by applicants making a submission to the EAC-MRH initiative

The top three challenges faced by applicants in making a submission to the EAC-MRH initiative were the lack of information on individual country or EAC websites about the submission process, milestones or timelines or a listing of pending and approved products (Figure 3). Further challenges include a lack of clarity about the process for submission and follow-up in each country, and the failure by countries to adhere to promised timelines.

Other challenges faced by pharmaceutical companies were the differences in time to the implementation of EAC recommendations by member countries; the risk of losing access to all member countries once a product is rejected by EAC-MRH as applicants can no longer pursue registration in individual countries and the need to update online submission and tracking by the applicant.

#### 4.4.2 Challenges faced by authorities in reviewing the EAC-MRH applications

Pharmaceutical companies stated several challenges faced by NMRAs in reviewing the EAC-MRH applications. It was claimed that the EAC-MRH requirements are more numerous and stringent as compared with those of individual countries, so companies need to provide all query details received from EAC. There are different levels of buy-in from individual countries and differing application requirements in some countries; for example, labelling requirements and some medicines are accepted in some countries but not others. The lack of legal/regulatory binding requirements in the national regulations is also a critical challenge and whilst some regional guidelines exist, they are not always fully implemented in the national regulations (Figure 3).

Another challenge is the lack of structured mechanisms for the execution of the joint assessment procedures, and limited capacity delays convening assessment meetings and eventually approvals. There are several logistical constraints including the lack of clear mandate between authorities and the EAC-MRH Secretariat, a lack of a permanent joint Secretariat and shared calendar that include NMRA schedules. Furthermore, the dependence on a single individual with sole responsibility for process at each authority is a key challenge. The coordination for good manufacturing process (GMP) inspections, including desk reviews and the sharing of information between countries was also mentioned as a challenge. The pharmaceutical companies commented that the lack of sustainable resources and funds dedicated to EAC-MRH affects the availability of assessors and the prioritisation of EAC-MRH assessment over national activities (Figure 3).

There is also a constraint in the flow of information among the active NMRAs who participate in the evaluation process, leading to a delay in adopting the recommendations from the outcome of the evaluation process by countries.

### 4.5 Part IV- improving performance (effectiveness and efficiency)

#### 4.5.1 Improving the effectiveness of the EAC initiative

**FIGURE 4:**
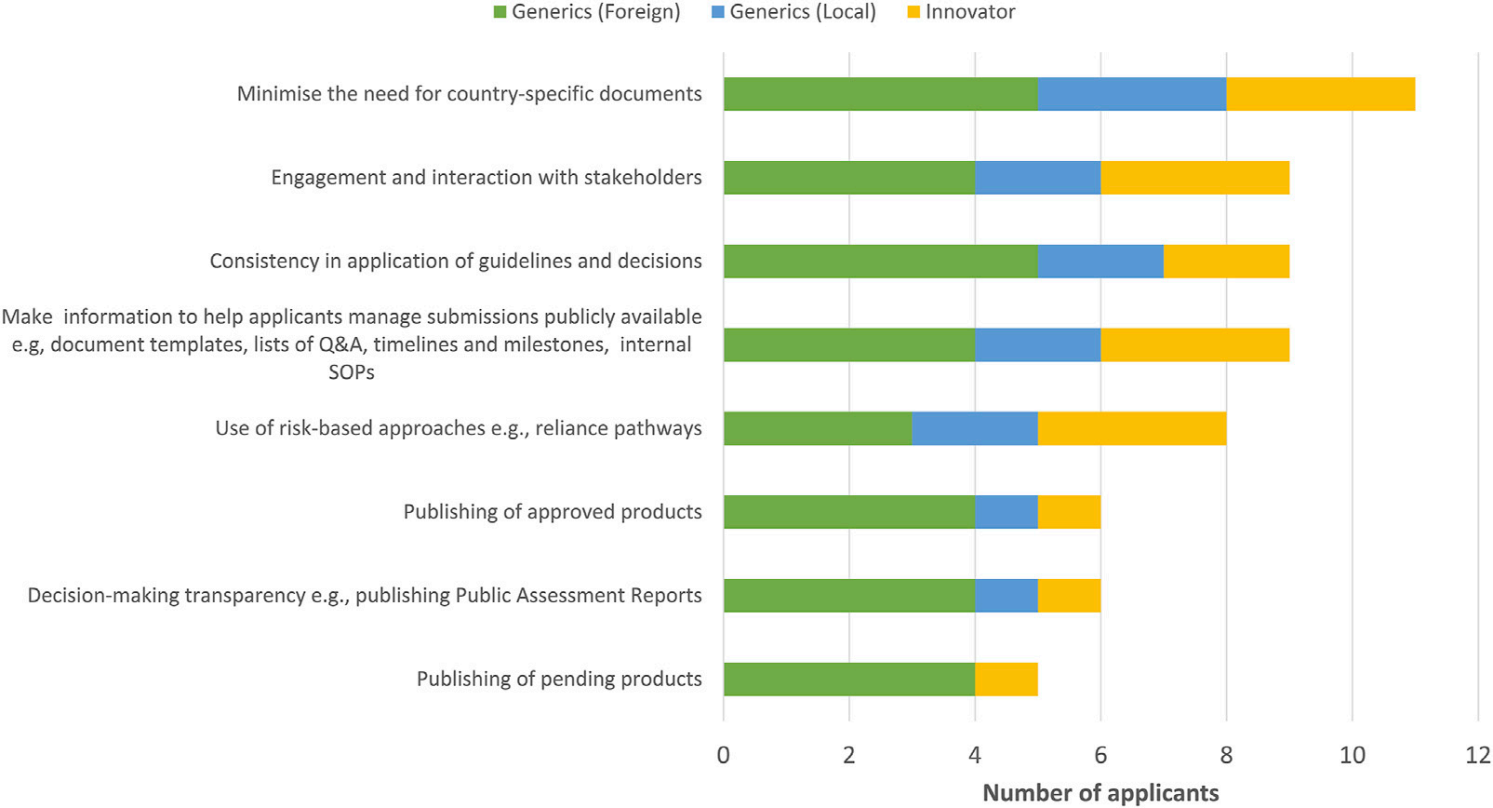
Ways to improve the effectiveness of the EAC initiative.

A number of ways to improve the effectiveness of the EAC initiative were mentioned, which include minimising the need for country-specific documents, engagement and interaction with stakeholders, making publicly available any information that might help applicants in managing their submissions such as document templates, lists of questions and answers, timelines and milestones, disclosure of internal standard operating procedures, consistency in application of guidelines and decisions and the use of risk-based approaches such as reliance pathways were identified by the majority of applicants as ways to improve effectiveness (Figure 4).

#### 4.5.2 Improving efficiency of the EAC-MRH initiative

**FIGURE 5:**
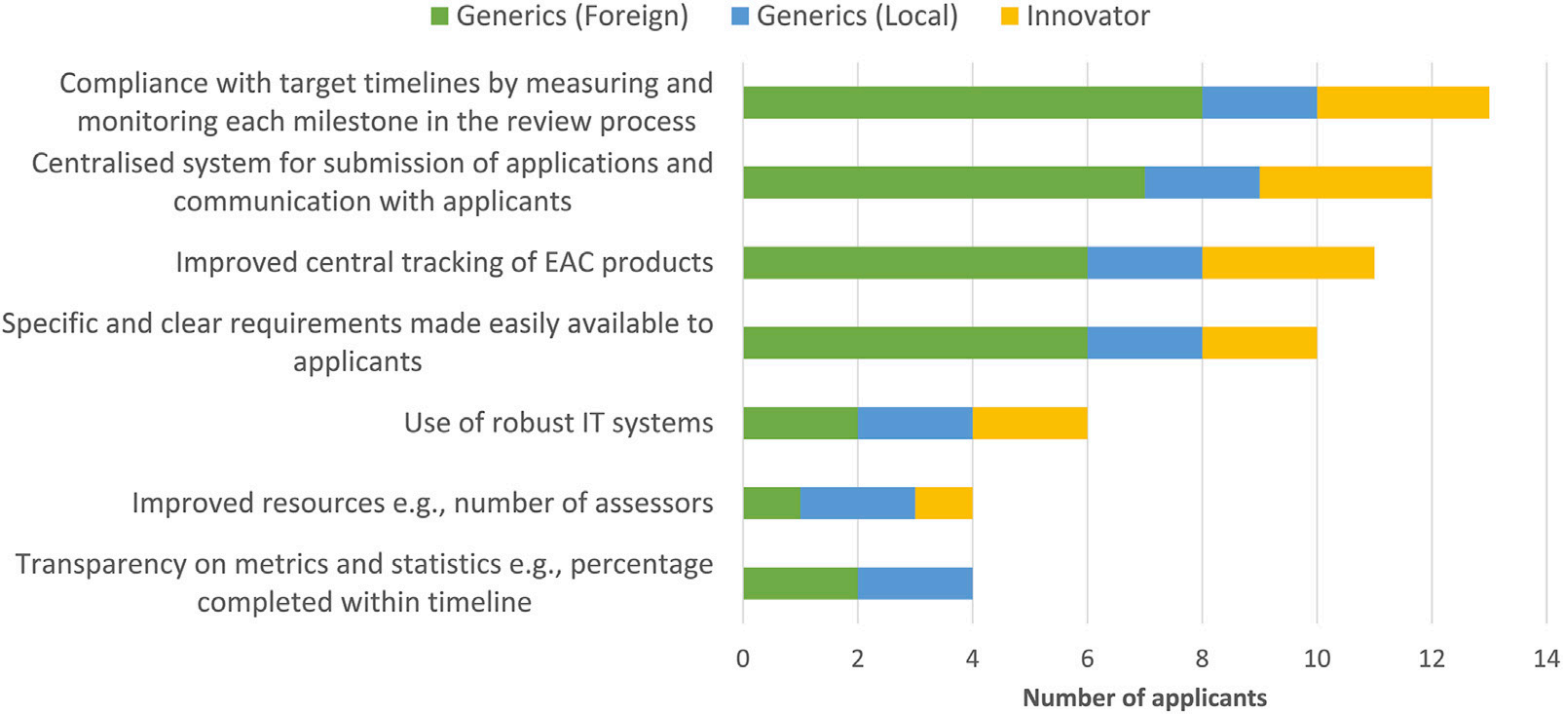
Ways to improve the efficiency of the EAC initiative.

Most applicants indicated that improving efficiency of the initiative would entail compliance with target timelines by measuring and monitoring each milestone in the review process (Figure 5). It would also include a centralised system for submission of applications and communication with applicants, improved central tracking of EAC products as well as specific and clear requirements made easily available to pharmaceutical companies. An appropriate regulatory framework that recognises and gives appropriate recognition and resources to regional procedures in national regulations would also be invaluable.

### 4.6 Part V–Strategies for improving the current EAC-MRH operating model

The main proposal made by the pharmaceutical companies to improve the EAC operating model is the establishment of a regional administrative body to centrally receive and track EAC applications. This approach would include being responsible for allocating work, apportioning the applicable fees to countries, tracking of applications and communication with applicants. The majority of the pharmaceutical companies were also of the view that the establishment of a Regional Medicines Authority in the EAC, if legally possible, would be the best strategy for improved performance.

Several reasons were given as to the importance, benefits and strengths of a regional authority and these included an established EAC centre with representatives/staff, which would avoid delays in the assessment process since the evaluation committee will be fully fledged instead of evaluators having to convene from various countries and/or regions. This would harmonise the registration process in the EAC partner states, leading to a less expensive and faster registration procedure. A regional authority would also improve access to medicines as it will enhance other interrelated aspects like the movement of goods, customs requirements as well as having just a license for the product may not be sufficiently efficient to assure product access.

Furthermore, a centralised review with legal responsibility to share reviews, documents, and activities between countries and the industry would minimise overlapping requests for inspections and information sharing. Centralising the evaluation process would increase the efficiency and effectiveness and make communication between stakeholders easier and clearer especially if there are dedicated personnel working in the regional medicines’ authority. Applicants would know exactly who to call and interact with regarding their submissions as the employees would only be involved with EAC applications and not applications from individual countries. Applicants also indicated that a regional authority would influence the development of an online portal for submission and tracking of the application status for the sponsors and also enable a faster and easier approval process with minimum requirements. The ease of verifying information centrally received for EAC-MRH applications would facilitate the tracking of applications and subsequent communication with the pharmaceutical companies.

However, some pharmaceutical companies were of the view that the establishment of a Regional Medicines Authority might not be a good strategy moving forward, especially if it encounters sustainability challenges where the authority has a higher workload and is underfunded. Another proposal was that with the ongoing activities by the African Union toward the operationalisation of the African Medicines Agency (AMA), there is now no additional need for duplication of regulatory processes with protracted lobbying times across the regions. The best approach would be to facilitate ongoing regional harmonisation frameworks and set the stage for a single Pan-African Agency (AMA). It is important to first clarify the EAC-MRH process, and the role of each individual NMRA, then to fully implement regional procedures in the national authorities. Adding a regional authority without solving the current challenges, would add to the complexity, especially considering that the continental authority (AMA) will soon be fully established. It would also become difficult for applicants to navigate between national, regional and continental institutions, as well as between numerous available registration pathways. Moreover, the challenge of lifecycle management, including post-approval changes submission/approval and license maintenance is still only foreseen by national procedures.

## 5 Discussion

The aim of this study was to evaluate the effectiveness and efficiency of the current operating model of the EAC-MRH initiative from the applicants’ perspective and to identify the challenges it faces as well as opportunities for improvement. Pharmaceutical companies affirmed the importance and relevance of the EAC-MRH work-sharing initiative, as it has benefitted regulators, applicants and patients in the region. As the first region to implement medicines regulatory harmonisation in Africa, the EAC has made major strides toward achieving its main objective of improving patients’ access to high-quality medicines in the region. The EAC-MRH initiative has made the process of registration and marketing authorisation more efficient to pharmaceutical companies through the use of harmonised technical standards and optimisation of regulatory requirements, thereby resulting in the reduction of timelines for review of applications (Mashingia et al., 2020; Ndomondo-Sigonda et al., 2020).

Comparing the views of applicants in this study with those of regulators Ngum et al. (2022), identified similar challenges. These included the lack of a centralised submission and tracking process for the work-sharing initiative entailing a lack of clarity about the process for submission and follow-up in each country for applicants. In addition, a lack of ability to mandate central registration has led to a failure by countries to adhere to promised timelines. The regional guidelines that exist are not fully implemented in all the countries. Furthermore, the unclear process for obtaining actual marketing authorisation after assessment through the initiative has caused various levels of company buy-in for the differing application requirements from individual countries. This delay by countries in issuing the actual market authorisation to applicants was affirmed in another study conducted in 2019 by Dansie and associates. The negative effect of the lack of information on individual country and EAC websites cannot be overemphasised and communication from the EAC Secretariat has also been lacking.

Moreover, due to limited capacity and resources, there is a weak coordination mechanism and the lack of structured mechanisms for the execution of the joint assessment procedures. This has led to the dependence of the initiative on the countries’ processes for communication with pharmaceutical companies and insufficient engagement between applicants/manufacturers and stakeholders. Finally, as reported by Dansie and others in 2019, the EAC-MRH initiative has not motivated increased company interest in country markets that are less attractive because of political or logistic issues.

### 5.1 Way forward

As a result of this study, it is recommended that there should be both effective communication and engagement by the industry with the agencies and coordinators should be empowered to talk directly with applicants. There should also be transparency in communication as well as adequate inclusion of all stakeholders, with the industry as a key user of the procedures in the relevant discussions. There should be predictability of processes and adherence to timelines and procedure. There is a need for a holistic approach for the EAC-MRH procedure in terms of eligible product categories and the inclusion of lifecycle management activities. Company study participants also suggested that financial incentives be given to applicants to follow the joint evaluation pathway; that is, fees for joint assessment should be lower when compared with those for single country assessment.

Adherence to the EAC-MRH process by the NMRAs should be promoted. Arik and others also recommended a cooperation framework agreement between NMRAs and the EAC (2020). Instituting a legally binding framework would enhance implementation of joint decisions (Giaquinto et al., 2020) and one of the study participants further suggested the elimination of national assessments of dossiers.

The following are some key recommendations to improve the effectiveness and efficiency of the EAC-MRH initiative.

• A study should be conducted to understand why the benefits of the work-sharing initiative have deteriorated over time in some countries and why a EAC positive opinion does not directly transform to individual country approvals.
• The EAC Secretariat should closely track national marketing authorisations and GMP assessments after a positive joint assessment to ensure that each country implements the registration within an appropriate timeframe.
• Financial incentives should be given to applicants to follow the joint evaluation pathways with the fees per country being lower for joint assessments compared with those for single country assessment.
• There is a need for engagement with the industry with a clear registration procedure for the EAC-MRH process. Clear guidance needs to be implemented based on established harmonised regulations and procedures across the whole region, and adhered to at the national level.
• Stronger mutual recognition is needed between member countries.
• The establishment of an EAC Regional Medicines Authority would be the best strategy for improved performance.

## 6 Conclusion

While harmonisation is key to ensuring access to safe, effective and high-quality medicines, there are also other elements of the healthcare system such as accessibility and affordability that need to be in place in order to realise the full benefits of the medicines regulatory harmonisation initiative. It is imperative for the recommendations made in this study to be fully implemented to ensure faster registration of the much-needed essential medicines by patients in the EAC region. Full implementation of the EAC road map 2020–2022 is critical to address some of the immediate issues. It is worth noting that Rwanda, one of the EAC member countries, will be hosting the African Medicines Agency and with the combined efforts by the African Union Partners to strengthen regulatory systems on the continent, the operationalisation of AMA would strengthen the EAC-MRH initiative.

## Data Availability

The raw data supporting the conclusions of this article will be made available by the authors, without undue reservation.

## Abbreviations

ABREMA, Burundi Food and Medicines Regulatory Authority, Republic of Burundi; AMA, African Medicines Agency; AU, African Union; CTD, Common Technical Document; DFCA, Drug and Food Control Authority, Republic of South Sudan; EAC, East African Community; Rwanda FDA, Rwanda Food and Drugs Authority, Republic of Rwanda; GMP, Good Manufacturing Practice; ICH, International Conference on Harmonisation; MRH, Medicines Regulatory Harmonization; NDA, National Drug Authority, Uganda; NMRAs, National Medical Regulatory Authorities; PPB, Pharmacy and Poisons Board, Republic of Kenya; RECs, regional economic communities; SAHPRA, South African Health Products Regulatory Authority; TMDA, Tanzania Medicines and Medical Devices Authority; TWG MER, Technical Working Group on Medicines Evaluation and Registration; WHO, World Health Organization; ZFDA, Zanzibar Food and Drug Medicines and Medical Devices Authority, the United Republic of Tanzania.
